# Brachial plexus nerve ultrasound in hereditary transthyretin amyloidosis

**DOI:** 10.1016/j.cnp.2026.10.006

**Published:** 2026-10-13

**Authors:** Chiara Briani, Mario Cacciavillani, Alessandro Salvalaggio

**Affiliations:** a Dept. of Neurosciences, Neurology Unit, University of Padova, Padova, Italy; b Neurologic Clinic Azienda Ospedale Università Padova, Italy; c CEMES-EMG Lab, Synlab Group, Padova, Italy

**Keywords:** Nerve nerve ultrasound, Brachial plexus, Hereditary transthyretin amyloidosis

Dear Editor,

We read with great interest the paper by Tawfik et al. entitled *“Neuromuscular ultrasound of the brachial plexus: know-how, know-when” (*Tawfik et al., 2026).

In their extensive and comprehensive review, the authors provide a rigorous analysis of the role of nerve ultrasound on the most common neurological plexopathies highlighting relevant anatomical, clinical, methodological and neurophysiological considerations.

We congratulate the authors on this thorough and carefully conducted review and would like to further enhance the importance of brachial plexus nerve ultrasound in clinical practice by sharing our experience in the diagnostic work-up and longitudinal assessment of patients with hereditary transthyretin amyloidosis (ATTRv, v as variant) (Salvalaggio et al., 2021).

With the aim of identifying a specific ultrasound morphological pattern capable of distinguishing idiopathic carpal tunnel syndrome (CTS) from ATTRv-associated CTS, we performed an extensive nerve ultrasound assessment in both ATTRv patients with polyneuropathy (ATTRv-PN) and pre-symptomatic carriers. We also investigated whether ultrasound parameters could serve as potential biomarkers for early disease detection.

The results of our study demonstrated that ATTRv-PN patients showed significantly larger nerve cross-sectional areas (CSAs) than pre-symptomatic carriers at several anatomical sites. Of particular interest are the findings at the level of the brachial plexus. Indeed, at the brachial plexus, the mean CSA was 97.5 mm^2^ (SD 25.2) in ATTRv-PN patients, compared with 65.5 mm^2^ (SD 16.2) in pre-symptomatic carriers (*p* < 0.001). Considering that nerve CSA usually does not increase in axonal neuropathies (Padua et al., 2018) and in some cases can even decrease (Salvalaggio et al., 2024),we thought that proximal nerve enlargement may represent a hallmark of ATTRv polyneuropathy and could potentially help distinguish ATTRv-PN from other axonal neuropathies. In a subsequent study, we demonstrated that morphological changes detected by ultrasound parallel disease progression, particularly at the level of the brachial plexus [Salvalaggio et al., 2022].

In conclusion, we agree with the authors that the brachial plexus can represent a “hot spot” requiring attention not only in disorders of the plexus (such as neuralgic amyotrophy) but also in other diseases of the peripheral nervous system.

## Declaration of competing interest

The authors declare that they have no known competing financial interests or personal relationships that could have appeared to influence the work reported in this paper.
